# Supplementary figures and images for: Structure–Activity Relationship of Novel ACE Inhibitory Undecapeptides from Stropharia rugosoannulata by Molecular Interactions and Activity Analyses
Source: Foods. 2023 Sep 17;12(18):3461. doi: 10.3390/foods12183461 (PMC10529921; doi:10.3390/foods12183461)

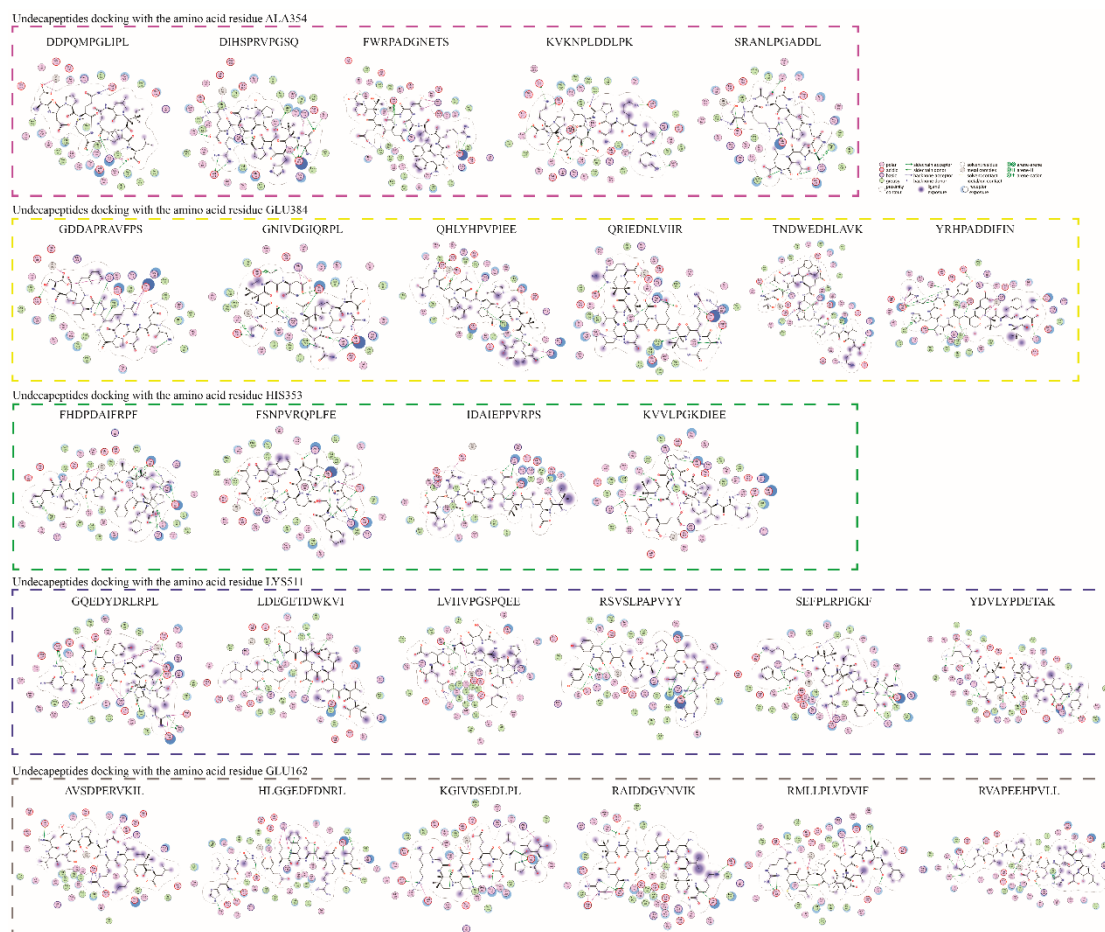

**Figure S1.** 2D interaction plots of the 27 *S. rugosoannulata* undecapeptides to the ACE receptors

Supplement: Supplementary file 1 [file foods-12-03461-s001.zip › Supplementary Figure S1.pdf]
